# Construction of a quality evaluation index system for waterless surgical hand antisepsis in operating rooms

**DOI:** 10.3389/fsurg.2026.1949101

**Published:** 2026-10-29

**Authors:** Songping Yang, Xi Yuan, Xuequn Yin, Wenjuan Zhou, Huixian Zhou, Li Miao, Guo Zhang, Xinmei Zhang

**Affiliations:** Department of Surgery, The First Affiliated Hospital of Soochow University, Suzhou, Jiangsu, China

**Keywords:** analytic hierarchy process, Delphi method, indicator system, operating room, quality evaluation, waterless surgical hand antisepsis

## Abstract

**Objective:**

To develop and obtain expert consensus on a quality evaluation index system for waterless surgical hand antisepsis in operating rooms and to assign hierarchical weights to the finalized indicators.

**Methods:**

Guided by the Donabedian structure-process-outcome model, candidate indicators were generated through a structured literature review and semi-structured interviews. A three-round expert consultation was conducted: Rounds 1 and 2 were used to screen and refine indicators, and Round 3 was used for AHP-based pairwise weighting. Twenty-two eligible experts received the Round-1 questionnaire; 20 returned valid questionnaires and constituted the panel retained for Rounds 2 and 3, in which all 20 responded. Expert authority was assessed using Cr = (Ca + Cs)/2. In Round 3, individual expert authority coefficients were normalized to obtain expert-specific weights, and pairwise judgments were aggregated using a weighted geometric mean before AHP weight calculation and consistency testing.

**Results:**

The valid response rates were 90.9% (20/22), 100% (20/20), and 100% (20/20) in Rounds 1, 2, and 3, respectively. The panel mean expert authority coefficients (Cr) were 0.883, 0.895, and 0.900. Kendall's coefficients of concordance for the two indicator-screening rounds were 0.242 and 0.334, respectively (both *P* < 0.001), while coefficients of variation decreased from 0.07-0.20 to 0.05-0.18. The aggregated AHP group judgment matrices satisfied the predefined consistency criterion (CR < 0.10). The final system comprised three first-level, nine second-level, and 28 third-level indicators. The global weights of the first-level domains were structure 0.1762, process 0.4123, and outcome 0.4115.

**Conclusion:**

This study produced a consensus-based and hierarchically weighted quality evaluation index system for waterless surgical hand antisepsis. The framework may support standardized quality assessment and future quality-improvement work; however, the present study establishes expert consensus and relative priorities rather than clinical effectiveness, psychometric reliability, or causal effects. Multicenter clinical validation is required before broad implementation.

## 1 Introduction

With the rapid development of healthcare services, the prevention and control of healthcare-associated infections have become an important component of medical quality and patient safety. Hand hygiene is a fundamental measure for reducing infection transmission and remains a major focus of infection-prevention programs worldwide (1). Surgical hand antisepsis is a key component of perioperative infection prevention because it aims to reduce transient and resident microorganisms on the hands and forearms of surgical personnel before sterile procedures (2, 3).

Surgical hand antisepsis can be performed using traditional scrub-based methods or alcohol-based waterless preparations. In a recent comparative study, mean bacterial counts decreased from 0.202 to 0.007 CFU/cm2 after conventional hand scrubbing (approximately 96.5%) and from 0.163 to 0.001 CFU/cm2 after alcohol-based hand rubbing (approximately 99.4%); however, the between-group difference was not statistically significant (*P* > 0.05) (4). Alcohol-based hand preparation may nevertheless provide operational advantages: reported preparation times were approximately 264 s for conventional scrubbing and 160 s for hand rubbing, while traditional water-based surgical scrubbing has been estimated to consume approximately 11 L of water per scrub (4, 5). These findings support comparable antimicrobial effectiveness while highlighting potential advantages in time and resource use. Nevertheless, adherence to recommended hand-antisepsis practices remains variable across clinical settings (6–8). Suboptimal technique may compromise infection-prevention efforts and therefore warrants systematic quality monitoring.

Existing regulations, professional standards, and technical guidelines provide important requirements for hand hygiene and surgical hand preparation. In parallel, individual healthcare institutions have developed local checklists or evaluation tools. However, published approaches frequently focus on selected procedural steps or single outcome measures, and their dimensions and definitions vary across institutions (9). A structured, multidimensional framework that links organizational prerequisites, implementation processes, and measurable outcomes may therefore be useful for standardized quality assessment and continuous improvement.

Accordingly, this study used the Donabedian structure-process-outcome (SPO) model (10) as the theoretical framework and combined a structured literature review, semi-structured interviews, Delphi expert consultation, and the Analytic Hierarchy Process. The aim was to develop a consensus-based quality evaluation index system for waterless surgical hand antisepsis in operating rooms and to estimate the relative priority of its indicators. The study was designed as an index-development study; clinical effectiveness and measurement performance in routine practice were not evaluated at this stage.

## 2 Methods

### 2.1 Establishment of the research team

The research team consisted of seven members: two operating room clinical nursing experts, one nursing management expert, one hospital infection management expert, two postgraduate nursing students, and one statistical expert. One operating room nursing expert, one nursing postgraduate student, and one hospital infection management expert with evidence-based practice training were responsible for literature retrieval, development of the preliminary item pool, and preparation of the Delphi questionnaire. The statistical expert and nursing postgraduate students were responsible for statistical analysis and data processing. The operating room nursing experts and nursing management expert were responsible for identifying eligible Delphi experts and organizing the consultation. All team members participated in indicator review, revision, and finalization.

### 2.2 Structured literature review and initial item generation

A structured literature review of both English-language and Chinese-language sources was undertaken to generate candidate indicators rather than to estimate an intervention effect. The following guideline repositories, evidence resources, and bibliographic databases were searched: UpToDate, BMJ, Guideline International Network (GIN), Scottish Intercollegiate Guidelines Network (SIGN), National Institute for Health and Care Excellence (NICE), New Zealand Guidelines Group (NZGG), Registered Nurses' Association of Ontario (RNAO), Association of Perioperative Registered Nurses (AORN), Agency for Healthcare Research and Quality (AHRQ), American Society of PeriAnesthesia Nurses (ASPAN), Arbeitsgemeinschaft der Wissenschaftlichen Medizinischen Fachgesellschaften (AWMF), CINAHL, Cochrane Library, Embase, Web of Science, PubMed, China National Knowledge Infrastructure (CNKI), Wanfang Database, VIP Database, and China Biomedical Literature Database. The search covered database inception through April 2025.

The search was based on common conceptual domains rather than a single identical literal search string across all resources. Search syntax was adapted to the indexing structure and search functions of individual databases and guideline repositories. The English search concepts included “surgical hand antisepsis”, “surgical hand preparation”, “presurgical hand preparation”, “surgical hand scrub*”, “presurgical scrub”, “hand antisepsis”, “hygienic hand antisepsis”, “hygienic handscrub”, “hygienic handwash”, “hand hygiene”, and “hand disinfection”, combined with “guideline”, “consensus”, “best practice recommendations”, and “recommended”. Chinese terms included “waterless surgical hand antisepsis”, “surgical hand antisepsis”, “preoperative hand antisepsis”, “hand hygiene”, “guideline”, “consensus”, “best practice”, “standard”, “quality evaluation”, “evaluation indicators”, and “indicator system”. The search concept framework is summarized in Supplementary Table S2.

A total of 112 records were identified. Three research-team members with training in evidence-based practice undertook the literature work. Two researchers independently screened the retrieved records for relevance to surgical hand antisepsis quality evaluation and assessed potentially eligible sources; disagreements were resolved through discussion, with the third researcher acting as adjudicator when consensus could not be reached. Sources were retained when they addressed surgical hand antisepsis or closely related perioperative hand-hygiene quality and contained recommendations, standards, implementation requirements, monitoring methods, quality indicators, or other information that could inform indicator development. Duplicate publications, records unrelated to surgical hand antisepsis quality evaluation, and publications without extractable indicator-related information were excluded. Eighteen sources were ultimately included. Candidate indicators were independently extracted, conceptually overlapping items were consolidated, and the resulting item pool was mapped to the Donabedian structure, process, and outcome domains (Figure 1).

**Figure 1:**
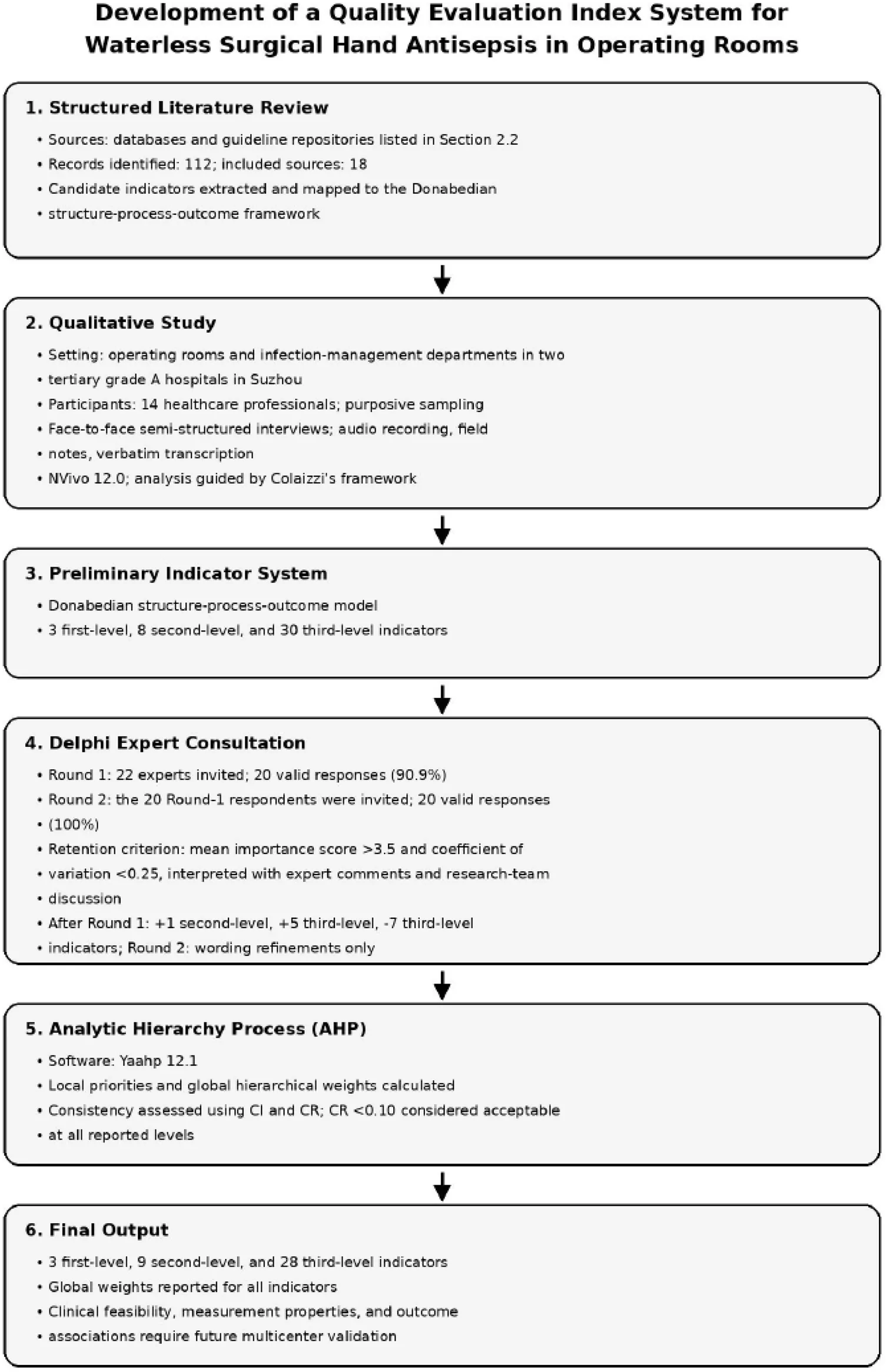
Flowchart of the development of the quality evaluation index system for waterless surgical hand antisepsis in operating rooms.

### 2.3 Semi-structured interviews

From May to June 2025, purposive sampling was used to recruit 14 healthcare professionals from operating rooms and hospital infection management departments of two tertiary grade A hospitals in Suzhou. Participants included operating room nursing staff, nursing managers, infection-management professionals, and surgeons, thereby providing perspectives from personnel involved in the implementation, supervision, management, and clinical use of surgical hand antisepsis. Professional titles ranged from junior to intermediate, associate senior, and senior levels, and professional experience ranged from 3 to 25 years.

The semi-structured interview guide addressed six domains: (1) the perceived need for a quality evaluation system and reasons for that view; (2) key concerns during waterless surgical hand antisepsis; (3) factors affecting standardized implementation; (4) aspects of current procedures requiring improvement; (5) clinically important dimensions for quality evaluation; and (6) additional suggestions. The complete interview guide, including the core questions posed to participants, is provided in Supplementary Table S1. Interviews were conducted face to face using the standardized guide, with each interview lasting approximately 20–30 min. With participants' permission, interviews were audio-recorded and supplemented by field notes. Recordings were transcribed verbatim and checked against the original audio for completeness and accuracy.

NVivo 12.0 was used to organize the qualitative data. Analysis was guided by Colaizzi's seven-step phenomenological framework (11). Transcripts were read repeatedly, statements relevant to quality problems and evaluation needs were identified, meanings were formulated and clustered, and related meanings were organized into themes. The thematic findings were compared with and used to supplement literature-derived candidate indicators. The combined item pool was then mapped to the Donabedian framework, yielding a preliminary system comprising three first-level, eight second-level, and 30 third-level indicators. Purposive sampling was used to obtain professional diversity across relevant stakeholder groups; because a prospectively documented thematic-saturation criterion was not used to determine sample size, saturation is not claimed as the stopping rule for recruitment.

### 2.4 Delphi expert consultation

#### 2.4.1 Expert eligibility, recruitment, and panel retention

Experts were purposively selected by the research team according to predefined eligibility criteria. Inclusion criteria were: (1) at least 10 years of experience in operating room nursing or a related field; (2) a bachelor's degree or higher; (3) an intermediate or senior professional title; (4) voluntary participation; and (5) willingness and ability to complete repeated consultation rounds. Potential experts who self-reported being “unfamiliar” or “very unfamiliar” with the topic were not considered eligible. Non-return of a questionnaire was treated as nonresponse rather than as an analytic exclusion.

Twenty-two eligible experts from 12 tertiary grade A hospitals in Jiangsu, Zhejiang, and Sichuan provinces received the Round-1 questionnaire. Twenty experts returned valid questionnaires and constituted the retained panel for Rounds 2 and 3; all 20 completed both subsequent rounds, and no replacement experts were added. The panel represented operating room clinical nursing, clinical medicine, nursing management and quality control, and hospital infection management. The panel was purposively constituted to obtain multidisciplinary and multi-institutional representation among professionals directly involved in surgical hand antisepsis and its quality management, with emphasis on relevant expertise and the feasibility of repeated participation across consultation rounds. Two of the 20 retained experts were members of the research team (one in operating room nursing management and one in hospital infection management); both met the same eligibility criteria, held senior professional titles, and had more than 15 years of relevant clinical experience. In accordance with the host institution's financial regulations, these two internal experts did not receive consultation fees.

#### 2.4.2 Development of the Delphi questionnaire

The Delphi questionnaire consisted of three sections. The first provided the study background, objectives, significance, and completion instructions. The second presented the hierarchical indicator system and asked experts to rate the importance of each indicator on a five-point Likert scale from 1 (“very unimportant”) to 5 (“very important”). Free-text fields allowed experts to recommend modification, addition, deletion, or clarification of indicators. The third section collected demographic information, familiarity with the topic, and the basis for expert judgment.

#### 2.4.3 Three-round consultation procedure and indicator-selection rules

Electronic questionnaires were used for all three rounds. In Rounds 1 and 2, experts rated the importance of each indicator on a five-point Likert scale and could provide free-text recommendations for modification, addition, deletion, or clarification. After each screening round, quantitative ratings and comments were collated and reviewed by the research team. Indicator decisions considered both the predefined quantitative criteria and the substantive content of expert comments and relevant evidence. Round 3 was conducted after the indicator set had been finalized and was used exclusively for AHP weighting; experts compared the relative importance of indicators within the same hierarchical level using pairwise comparisons.

The predefined quantitative retention criteria for Rounds 1 and 2 were a mean importance score >3.5 and a coefficient of variation (CV) < 0.25 (12). These thresholds were used as decision aids rather than as the sole basis for retention; additions, deletions, and wording changes were finalized through research-team discussion after considering expert comments and the supporting literature. After Round 1, indicators were added, deleted, and revised. In Round 2, no indicators were added or deleted and only descriptive wording was refined; the indicator set was therefore considered stable and was carried forward to Round 3 for weight assignment.

### 2.5 Data collection and data management

Data collection was conducted by three research-team members who had received training in qualitative research methods and evidence-based nursing. One operating room nursing expert and one nursing master's student conducted the semi-structured interviews, while one hospital infection management expert coordinated the Delphi consultation.

For the Delphi phase, completed electronic questionnaires were exported and independently checked by two researchers for completeness and consistency before analysis. Responses were anonymized and assigned unique codes. Interview transcripts, audio recordings, field notes, and Delphi datasets were stored on password-protected computers accessible only to the research team and backed up on encrypted institutional storage. Personal identifiers were removed before analysis, and research materials were managed in accordance with institutional data-security and confidentiality requirements.

### 2.6 Statistical analysis and analytic hierarchy process

Data were analyzed using Microsoft Excel 2022 and SPSS version 27.0. Categorical variables are reported as frequencies and percentages, and continuous variables as mean ± standard deviation. Expert participation was summarized using valid questionnaire response rates. For Rounds 1 and 2, the concentration of expert ratings was described using mean importance scores and dispersion was assessed using the coefficient of variation (CV). Inter-expert agreement in the two screening rounds was assessed using Kendall's coefficient of concordance (W), with statistical significance set at *α* = 0.05. Kendall's W was not used for Round 3 because that round consisted of pairwise AHP judgments rather than Likert importance ratings.

Expert authority was assessed using the authority coefficient (Cr). For expert k, Cr_k = (Ca_k + Cs_k)/2, where Ca_k is the judgment-basis coefficient and Cs_k is the familiarity coefficient (13). The panel mean Cr values for Rounds 1, 2, and 3 were 0.883, 0.895, and 0.900, respectively. These coefficients describe the expertise of the consultation panel and were not interpreted as evidence of instrument reliability or clinical validity. For the Round-3 AHP group decision, the individual Cr_k values were further used to derive normalized expert-specific aggregation weights.

After the indicator set was finalized, Round 3 was used to determine relative weights by the Analytic Hierarchy Process (AHP) in Yaahp version 12.1. The hierarchy comprised the overall evaluation goal, the three first-level structure-process-outcome domains, their second-level dimensions, and the corresponding third-level indicators. Each of the 20 experts independently performed pairwise comparisons of indicators within the same hierarchical level using Saaty's 1–9 scale, generating reciprocal judgment matrices. To account for differences in expert authority, expert-specific weights were calculated by normalizing the individual authority coefficients: w_k = Cr_k/*Σ*Cr_k, with *Σ*w_k = 1. Individual pairwise judgments were then aggregated using the weighted geometric mean method. For each comparison between indicators i and j, the group judgment was calculated as a_ij = *Π*[(a_ij^(k))^(w_k)], where a_ij^(k) denotes the judgment provided by expert k and w_k denotes that expert's normalized authority weight. The aggregated values formed a group judgment matrix for each hierarchical level.

Yaahp 12.1 was used to derive priority vectors and normalized local weights from these group matrices, and global weights were obtained by multiplying local weights through the hierarchy. Consistency was evaluated using CI = (*λ*max - n)/(n - 1) and CR = CI/RI, where *λ*max is the maximum eigenvalue, n is the matrix order, and RI is the random consistency index. A CR < 0.10 was considered acceptable (12). The group judgment matrices used for final weighting satisfied this criterion. The consistency assessment evaluates the logical coherence of pairwise judgments and was not interpreted as evidence of clinical validity. The analyses in this study were intended to describe expert ratings, quantify expert agreement, and derive relative indicator priorities; clinical-effectiveness and psychometric testing were outside the scope of this index-development study.

### 2.7 Ethical considerations

This study was conducted in accordance with the Declaration of Helsinki and relevant national regulations. Ethical approval was obtained from the Ethics Committee of The First Affiliated Hospital of Soochow University (Approval No. 2025968). All healthcare professionals participating in the semi-structured interviews and all Delphi experts were informed of the study objectives and procedures, and written informed consent was obtained. Participation was voluntary, and participants could withdraw at any time without adverse consequences. Confidentiality and anonymity were maintained throughout the study.

## 3 Results

### 3.1 Expert recruitment and characteristics

Twenty-two eligible experts received the first-round questionnaire. Twenty returned valid questionnaires (90.9%) and formed the retained panel for Rounds 2 and 3; all 20 completed both subsequent rounds (100% in each round). Thus, the demographic characteristics reported below refer to the 20 experts who participated in the complete three-round consultation.

Among the 20 experts, four were operating room clinical nursing experts, five were clinical medical experts, ten were nursing management and quality control experts, and one was a hospital infection management expert. The experts were 33–54 years old (45.00 ± 6.58 years) and had 10–35 years of professional experience (21.45 ± 7.53 years). Fourteen held bachelor's degrees, two held master's degrees, and four held doctoral degrees. Seventeen held senior professional titles and three held intermediate titles.

### 3.2 Expert participation and authority

In Round 1, 22 questionnaires were distributed and 20 valid questionnaires were returned; eight respondents provided free-text modification suggestions. In Round 2, 20 questionnaires were distributed and all 20 were returned; three respondents provided further suggestions. In Round 3, 20 AHP weighting questionnaires were distributed and all 20 were returned. The corresponding valid response rates were 90.9%, 100%, and 100%. The panel mean expert authority coefficients (Cr) were 0.883, 0.895, and 0.900 for Rounds 1, 2, and 3, respectively (Table 1). Individual Round-3 authority coefficients were normalized to obtain expert-specific weights for the AHP group aggregation. The Cr values describe expert-panel expertise and should not be interpreted as evidence of instrument reliability or clinical validity.

**Table 1:** Expert participation and authority across the three consultation rounds.

Item	Round 1	Round 2	Round 3
Questionnaires distributed	22	20	20
Valid questionnaires returned	20	20	20
Response rate (%)	90.9	100	100
Experts providing suggestions	8	3	-
Panel mean authority coefficient (Cr)	0.883	0.895	0.900

Cr values shown in Table 1 are panel-level mean authority coefficients. For Round 3, each expert's individual authority coefficient (Cr_k) was normalized and used as the expert-specific weight in the weighted geometric aggregation of AHP pairwise judgments.

### 3.3 Expert consensus and coordination

For the two indicator-screening rounds, the CVs of importance ratings ranged from 0.07 to 0.20 in Round 1 and from 0.05 to 0.18 in Round 2. Kendall's W increased from 0.242 to 0.334, and both tests were statistically significant (*P* < 0.001) (Table 2). These findings indicate statistically significant and improving agreement across the two screening rounds, although the magnitude of W is more appropriately interpreted as modest-to-moderate concordance rather than high consistency. Round 3 was an AHP weighting round; the aggregated group judgment matrices used for final weighting had CR values < 0.10, indicating acceptable logical consistency of the pairwise judgments.

**Table 2:** Expert consensus and coordination.

Indicator	Round 1	Round 2
Coefficient of variation	0.07–0.20	0.05–0.18
Kendall's coefficient of concordance (W)	0.242	0.334
*P* value	<0.001	<0.001

### 3.4 Indicator refinement and AHP weighting

The preliminary framework contained three first-level, eight second-level, and 30 third-level indicators. After Round 1, one second-level indicator (“standardized operation supervision”) and five third-level indicators were added, seven third-level indicators were removed, and ten indicator labels or descriptions were revised. The net result after Round 1 was therefore three first-level, nine second-level, and 28 third-level indicators. Examples of wording refinement included changing “correct rate of operation sequence” to “correct operation procedure rate.”

In Round 2, no indicators were added or deleted; revisions were limited to descriptive wording. For example, “compliance rate of handwashing hardware” was changed to “hand antisepsis infrastructure compliance rate,” and “achievement rate of visual guidance and environmental cleaning maintenance” was changed to “visualization and environmental support rate.” The final system comprised three first-level, nine second-level, and 28 third-level indicators.

In Round 3, the 20 experts independently completed pairwise comparisons of the finalized indicators. Individual expert authority coefficients were normalized to obtain expert-specific aggregation weights, and the pairwise judgments were synthesized using the weighted geometric mean method to construct group judgment matrices. All group judgment matrices used for final weighting satisfied the predefined consistency criterion (CR < 0.10). The resulting global weights of the first-level domains were 0.1762 for structure, 0.4123 for process, and 0.4115 for outcome. Table 3 presents the final indicator set, Round-2 importance scores and CVs, and the global AHP weights derived from Round 3.

**Table 3:** Quality evaluation index system and global weights for waterless surgical hand antisepsis in operating rooms.

Indicator	Importance score (mean ± SD)	Coefficient of variation	Global weight
1. Structure	**4.55** ± **0.51**	**0**.**11**	**0**.**1762**
1.1 Facility configuration and utilization	**4.55** ± **0.51**	**0**.**11**	**0**.**0231**
1.1.1 Hand antisepsis infrastructure compliance rate	4.54 ± 0.58	0.13	0.0126
1.1.2 Visualization and environmental support rate	4.25 ± 0.57	0.14	0.0105
1.2 Position allocation and personnel management	**4.45** ± **0.60**	**0**.**14**	**0**.**0255**
1.2.1 Qualification rate of training and assessment personnel	4.50 ± 0.61	0.13	0.0092
1.2.2 Qualification rate of supervision and management personnel	4.55 ± 0.51	0.11	0.0107
1.2.3 Responsibility implementation rate of supporting personnel	4.30 ± 0.73	0.17	0.0056
1.3 Institutional standards and regulations	**4.65** ± **0.59**	**0**.**13**	**0**.**0638**
1.3.1 Completeness rate of standard operating procedures (SOPs) for surgical hand antisepsis	4.80 ± 0.52	0.11	0.0197
1.3.2 Completeness rate of surgical hand antisepsis-related institutional systems	4.65 ± 0.59	0.13	0.0192
1.3.3 Awareness rate of key contents of surgical hand antisepsis	4.75 ± 0.44	0.09	0.0249
1.4 Training and assessment	**4.75** ± **0.44**	**0**.**09**	**0**.**0638**
1.4.1 Standardization rate of surgical hand antisepsis training contents	4.60 ± 0.50	0.11	0.0127
1.4.2 Implementation rate of surgical hand antisepsis training	4.70 ± 0.47	0.10	0.0242
1.4.3 Qualification rate of surgical hand antisepsis assessment	4.70 ± 0.47	0.10	0.0269
2. Process	**4.85** ± **0.37**	**0**.**08**	**0**.**4123**
2.1 Pre-operation assessment and preparation	**4.40** ± **0.50**	**0**.**11**	**0**.**0546**
2.1.1 Environmental assessment implementation rate	4.30 ± 0.57	0.13	0.0080
2.1.2 Hand cleanliness and skin condition qualification rate	4.55 ± 0.51	0.11	0.0175
2.1.3 Nail standardization rate	4.50 ± 0.51	0.11	0.0168
2.1.4 Dressing and protection compliance rate	4.50 ± 0.51	0.11	0.0123
2.2 Operation implementation	**4.85** ± **0.37**	**0**.**08**	**0**.**1963**
2.2.1 Correct operation procedure rate	4.75 ± 0.44	0.09	0.0264
2.2.2 Correct application rate of antiseptic agent	4.90 ± 0.31	0.06	0.0496
2.2.3 Complete coverage rate of antiseptic agent	4.85 ± 0.37	0.08	0.0588
2.2.4 Compliance rate of operation duration	4.75 ± 0.44	0.09	0.0388
2.2.5 Compliance rate of operation posture	4.45 ± 0.94	0.21	0.0227
2.3 Standardized operation supervision	**4.55** ± **0.51**	**0**.**11**	**0**.**1613**
2.3.1 Real-time supervision implementation rate	4.35 ± 0.49	0.11	0.0415
2.3.2 Implementation rate of corrective actions for improper operations	4.65 ± 0.49	0.11	0.0666
2.3.3 Effect monitoring implementation rate	4.65 ± 0.49	0.11	0.0532
3. Outcome	**4.75** ± **0.44**	**0**.**09**	**0**.**4115**
3.1 Clinical outcomes	**4.90** ± **0.31**	**0**.**06**	**0**.**2264**
3.1.1 Qualification rate of biological monitoring	4.80 ± 0.41	0.09	0.0895
3.1.2 Compliance rate of surgical hand antisepsis procedures	4.70 ± 0.47	0.10	0.1369
3.2 Effect evaluation	**4.65** ± **0.59**	**0**.**13**	**0**.**1851**
3.2.1 Quality feedback implementation rate	4.60 ± 0.60	0.13	0.0523
3.2.2 Quality problem improvement rate	4.55 ± 0.51	0.11	0.0842
3.2.3 Operator satisfaction	4.25 ± 0.55	0.13	0.0486

## 4 Discussion

### 4.1 Rationale and potential use of the quality evaluation framework

Quality evaluation of waterless surgical hand antisepsis is often centered on direct observation of technique or selected process measures, whereas implementation also depends on organizational prerequisites, supervision, feedback, and measurable outcomes. The present study therefore used the Donabedian framework to organize indicators into structure, process, and outcome domains. This approach provides a transparent way to connect environmental and organizational conditions with implementation behaviors and quality-management outcomes (10, 14).

The structural domain addresses facility configuration, personnel management, institutional standards, and training. The process domain includes pre-operation assessment and preparation, execution of the antisepsis procedure, and standardized supervision. The outcome domain includes biological monitoring, procedural compliance, feedback, improvement of identified problems, and operator satisfaction. Taken together, these domains offer a broader framework than a single procedural checklist and may support consistent data collection and quality-improvement planning.

The proposed index system should, however, be viewed as a development-stage framework rather than a clinically validated instrument. The present study did not test whether use of the system improves surgical-site infection rates, compliance, resource use, or other patient outcomes. Its immediate contribution is to define candidate quality dimensions and expert-derived priorities that can be evaluated prospectively in clinical practice. The framework may also inform future work on digital or intelligent monitoring, but such applications require separate technical and clinical validation (15–17).

### 4.2 Methodological rigor, expert consensus, and limitations of consensus evidence

The indicator-development process combined evidence review, qualitative input from healthcare professionals, Delphi consultation, and hierarchical weighting. These methods provide complementary forms of content development: the literature contributes evidence and standards, interviews identify practice-based concerns, Delphi rounds refine content through structured expert feedback, and AHP expresses relative priorities numerically. This design supports methodological transparency and content organization but does not by itself establish psychometric reliability, criterion validity, or clinical effectiveness.

The retained panel comprised 20 experts who completed all three rounds and represented operating room nursing, clinical medicine, nursing management and quality control, and hospital infection management. All had at least 10 years of relevant experience and held intermediate or senior professional titles. The valid response rates were 90.9%, 100%, and 100% across the three rounds. Kendall's W increased from 0.242 to 0.334 between the two indicator-screening rounds; both tests were statistically significant (*P* < 0.001), but the magnitude of W indicates only modest-to-moderate concordance rather than strong consensus. In the third round, individual expert authority coefficients were normalized to define expert-specific contributions, and AHP pairwise judgments were combined using a weighted geometric mean before derivation of the group weights; the resulting group judgment matrices satisfied the prespecified CR < 0.10 criterion. The CR criterion reflects the internal logical consistency of pairwise judgments and does not establish measurement accuracy or clinical validity.

Two retained experts were research-team members; although both met the same eligibility requirements as external experts, their inclusion may have introduced a degree of investigator-allegiance bias. This should be considered when interpreting the consensus results and reinforces the need for external multicenter validation.

The panel was drawn from 12 tertiary grade A hospitals in three Chinese provinces, and the qualitative interviews were conducted in two hospitals in Suzhou. The resulting indicators may therefore reflect the organization, terminology, and priorities of these settings. Broader geographic and institutional validation is needed before claiming generalizability. In addition, expert authority coefficients, CVs, and Kendall's W are measures of panel characteristics and consensus; they should not be interpreted as evidence that the index system is reliable, objective, accurate, or valid in routine clinical measurement. The present study did not evaluate inter-rater reliability, criterion or construct validity, responsiveness, or associations with clinical outcomes.

Although the multidisciplinary panel was intentionally constituted to cover clinical practice, operating-room nursing, quality management, and infection prevention, it was not designed to represent the occupational distribution of all end users of surgical hand antisepsis. Professionals who directly perform surgical hand preparation, particularly surgeons, should be more extensively represented in future multicenter validation. Stratified evaluation across surgeons, operating-room nurses, infection-prevention personnel, and quality-management professionals may help determine whether the perceived importance, feasibility, and operational interpretation of individual indicators differ among stakeholder groups.

### 4.3 Interpretation of indicator weights

The first-level global weights were 0.4123 for process, 0.4115 for outcome, and 0.1762 for structure. Thus, the expert panel assigned similar relative priority to process and outcome indicators and a lower relative priority to structural indicators. These AHP weights represent expert-derived priorities within the constructed hierarchy; they are not effect sizes and should not be interpreted as evidence that one domain has a greater causal influence on infection outcomes than another.

Within the structural domain, institutional standards and regulations and training and assessment received the largest global weights (both 0.0638). This pattern is consistent with literature emphasizing standardized procedures, education, supervision, and sustained quality-improvement mechanisms in hand hygiene and surgical hand preparation (18–22). The relatively small weight assigned to supporting-personnel responsibility implementation (0.0056) indicates lower relative priority within this panel, not lack of clinical relevance.

Within the process domain, operation implementation (0.1963) and standardized operation supervision (0.1613) received the highest global weights. Complete antiseptic coverage, correct application, adequate duration, and corrective action after improper technique were prioritized by experts. Evidence syntheses and international guidance similarly emphasize standardized technique and adherence as important components of surgical hand preparation and infection prevention (23–25). The present study did not test whether the higher-weighted process indicators independently reduce surgical-site infection risk.

Within the outcome domain, procedural compliance (0.1369) had a higher global weight than biological monitoring qualification (0.0895), while quality problem improvement (0.0842) received the largest weight within effect evaluation. These priorities are compatible with a continuous-quality-improvement perspective, in which measurement is linked to feedback and corrective action. Nevertheless, the relative weights should be regarded as provisional until their feasibility, inter-rater reproducibility, sensitivity to quality change, and associations with clinically meaningful outcomes are examined in multicenter applications.

Overall, the Delphi and AHP results provide evidence of expert consensus and a structured prioritization scheme (26–31). They do not establish construct validity, predictive validity, or causal pathways. Future studies should therefore define operational numerators and denominators for each indicator, pilot the system in heterogeneous operating rooms, evaluate data-collection burden and inter-rater reliability, assess responsiveness to quality-improvement interventions, and examine associations with microbiological and patient-safety outcomes.

## 5 Conclusion

Using the Donabedian model, a structured literature review, semi-structured interviews, two indicator-screening Delphi rounds, and a third AHP weighting round, this study developed a quality evaluation index system for waterless surgical hand antisepsis comprising three first-level, nine second-level, and 28 third-level indicators. The study provides a transparent, consensus-based framework and relative weighting scheme that may support standardized assessment and quality-improvement planning in operating rooms.

Because the system has not yet undergone multicenter clinical testing, its feasibility, measurement reliability, validity, responsiveness, and relationship with clinical outcomes remain to be established. Future multicenter studies should refine operational definitions, test the indicators in routine practice, include broader representation of direct users such as surgeons, and evaluate whether use of the framework contributes to more consistent hand-antisepsis practice and better infection-prevention performance.

## Data Availability

The original contributions presented in the study are included in the article/Supplementary Material, further inquiries can be directed to the corresponding author.
