# Supplementary material for: Construction of a quality evaluation index system for waterless surgical hand antisepsis in operating rooms
Source: Front Surg. 2026 Oct 29;13:1949101. doi: 10.3389/fsurg.2026.1949101 (PMC13755290; doi:10.3389/fsurg.2026.1949101)
Supplement: Supplementary file 1 [file Datasheet1.docx]

| Item | Round 1 | Round 2 | Round 3 |
| --- | --- | --- | --- |
| Questionnaires distributed | 22 | 20 | 20 |
| Valid questionnaires returned | 20 | 20 | 20 |
| Response rate (%) | 90.9 | 100 | 100 |
| Experts providing suggestions | 8 | 3 | — |
| Panel mean authority coefficient (Cr) | 0.883 | 0.895 | 0.900 |

*Table 1. Expert participation and authority across the three consultation rounds.*

| Indicator | Round 1 | Round 2 |
| --- | --- | --- |
| Coefficient of variation | 0.07-0.20 | 0.05-0.18 |
| Kendall's coefficient of concordance (W) | 0.242 | 0.334 |
| P value | <0.001 | <0.001 |

*Table 2. Expert consensus and coordination*

| Indicator | Importance score (mean ± SD) | Coefficient of variation | Global weight |
| --- | --- | --- | --- |
| **1. Structure** | **4.55±0.51** | **0.11** | **0.1762** |
| **1.1 Facility configuration and utilization** | **4.55±0.51** | **0.11** | **0.0231** |
| 1.1.1 Hand antisepsis infrastructure compliance rate | 4.54±0.58 | 0.13 | 0.0126 |
| 1.1.2 Visualization and environmental support rate | 4.25±0.57 | 0.14 | 0.0105 |
| **1.2 Position allocation and personnel management** | **4.45±0.60** | **0.14** | **0.0255** |
| 1.2.1 Qualification rate of training and assessment personnel | 4.50±0.61 | 0.13 | 0.0092 |
| 1.2.2 Qualification rate of supervision and management personnel | 4.55±0.51 | 0.11 | 0.0107 |
| 1.2.3 Responsibility implementation rate of supporting personnel | 4.30±0.73 | 0.17 | 0.0056 |
| **1.3 Institutional standards and regulations** | **4.65±0.59** | **0.13** | **0.0638** |
| 1.3.1 Completeness rate of standard operating procedures (SOPs) for surgical hand antisepsis | 4.80±0.52 | 0.11 | 0.0197 |
| 1.3.2 Completeness rate of surgical hand antisepsis-related institutional systems | 4.65±0.59 | 0.13 | 0.0192 |
| 1.3.3 Awareness rate of key contents of surgical hand antisepsis | 4.75±0.44 | 0.09 | 0.0249 |
| **1.4 Training and assessment** | **4.75±0.44** | **0.09** | **0.0638** |
| 1.4.1 Standardization rate of surgical hand antisepsis training contents | 4.60±0.50 | 0.11 | 0.0127 |
| 1.4.2 Implementation rate of surgical hand antisepsis training | 4.70±0.47 | 0.10 | 0.0242 |
| 1.4.3 Qualification rate of surgical hand antisepsis assessment | 4.70±0.47 | 0.10 | 0.0269 |
| **2. Process** | **4.85±0.37** | **0.08** | **0.4123** |
| **2.1 Pre-operation assessment and preparation** | **4.40±0.50** | **0.11** | **0.0546** |
| 2.1.1 Environmental assessment implementation rate | 4.30±0.57 | 0.13 | 0.0080 |
| 2.1.2 Hand cleanliness and skin condition qualification rate | 4.55±0.51 | 0.11 | 0.0175 |
| 2.1.3 Nail standardization rate | 4.50±0.51 | 0.11 | 0.0168 |
| 2.1.4 Dressing and protection compliance rate | 4.50±0.51 | 0.11 | 0.0123 |
| **2.2 Operation implementation** | **4.85±0.37** | **0.08** | **0.1963** |
| 2.2.1 Correct operation procedure rate | 4.75±0.44 | 0.09 | 0.0264 |
| 2.2.2 Correct application rate of antiseptic agent | 4.90±0.31 | 0.06 | 0.0496 |
| 2.2.3 Complete coverage rate of antiseptic agent | 4.85±0.37 | 0.08 | 0.0588 |
| 2.2.4 Compliance rate of operation duration | 4.75±0.44 | 0.09 | 0.0388 |
| 2.2.5 Compliance rate of operation posture | 4.45±0.94 | 0.21 | 0.0227 |
| **2.3 Standardized operation supervision** | **4.55±0.51** | **0.11** | **0.1613** |
| 2.3.1 Real-time supervision implementation rate | 4.35±0.49 | 0.11 | 0.0415 |
| 2.3.2 Implementation rate of corrective actions for improper operations | 4.65±0.49 | 0.11 | 0.0666 |
| 2.3.3 Effect monitoring implementation rate | 4.65±0.49 | 0.11 | 0.0532 |
| **3. Outcome** | **4.75±0.44** | **0.09** | **0.4115** |
| **3.1 Clinical outcomes** | **4.90±0.31** | **0.06** | **0.2264** |
| 3.1.1 Qualification rate of biological monitoring | 4.80±0.41 | 0.09 | 0.0895 |
| 3.1.2 Compliance rate of surgical hand antisepsis procedures | 4.70±0.47 | 0.10 | 0.1369 |
| **3.2 Effect evaluation** | **4.65±0.59** | **0.13** | **0.1851** |
| 3.2.1 Quality feedback implementation rate | 4.60±0.60 | 0.13 | 0.0523 |
| 3.2.2 Quality problem improvement rate | 4.55±0.51 | 0.11 | 0.0842 |
| 3.2.3 Operator satisfaction | 4.25±0.55 | 0.13 | 0.0486 |

*Table 3. Quality evaluation index system and global weights for waterless surgical hand antisepsis in operating*

Figure 1. Flowchart of the development of the quality evaluation index system for waterless surgical hand antisepsis in operating rooms.

# Supplementary Materials

**Supplementary Table S1. Semi-structured interview guide**

| **Domain** | **Core question posed to participants** |
| --- | --- |
| Need for a quality evaluation system | Do you think it is necessary to establish a quality evaluation system specifically for waterless surgical hand antisepsis? Why or why not? |
| Key concerns in practice | In your clinical experience, what aspects or problems during waterless surgical hand antisepsis require the greatest attention? |
| Factors affecting standardized implementation | What factors do you think influence the standardized implementation of waterless surgical hand antisepsis in operating-room practice? |
| Areas requiring improvement | Which aspects of the current surgical hand-antisepsis process do you think require improvement, and why? |
| Clinically important evaluation dimensions | Which dimensions or specific elements do you consider most important when evaluating the quality of waterless surgical hand antisepsis? |
| Additional suggestions | Are there any other indicators, concerns, or suggestions that should be considered when constructing the quality evaluation system? |

**Note:** Follow-up probes were used as appropriate to clarify responses and elicit practical examples without changing the core interview domains.

**Supplementary Table S2. Search concept framework**

| **Component** | **English-language sources** | **Chinese-language sources** |
| --- | --- | --- |
| Primary concept | surgical hand antisepsis; surgical hand preparation; presurgical hand preparation; surgical hand scrub*; presurgical scrub; hand antisepsis; hygienic hand antisepsis; hygienic handscrub; hygienic handwash; hand hygiene; hand disinfection | waterless surgical hand antisepsis; surgical hand antisepsis; preoperative hand antisepsis; hand hygiene |
| Evidence / guidance concept | guideline; consensus; best practice recommendations; recommended | guideline; consensus; best practice; standard |
| Quality-evaluation concept | quality evaluation; evaluation indicators; indicator system (used where relevant to the resource) | quality evaluation; evaluation indicators; indicator system |
| Implementation note | The same conceptual domains were retained across resources; syntax was adapted to the indexing structure and search functions of each database or guideline repository. | The same conceptual domains were retained across resources; Chinese terminology and syntax were adapted to the corresponding database or repository. |

**Note:** The review was conducted to generate candidate indicators rather than to estimate an intervention effect. The table therefore reports the search concept framework and does not imply that one identical literal search string was applied across all resources.
